# The Rising Burden of Cardiovascular Disease and Thrombosis in India: An Epidemiological Review

**DOI:** 10.7759/cureus.73786

**Published:** 2024-11-15

**Authors:** Linthoingambi Loitongbam, William R Surin

**Affiliations:** 1 Amity Institute of Public Health and Hospital Administration, Amity University, Noida, IND; 2 Department of Microbiology and Cell Biology, Indian Institute of Science, Bangalore, IND

**Keywords:** cardiovascular disease, disease burden, hypertension, intravascular thrombosis, mental health

## Abstract

Cardiovascular disease (CVD) associated with intravascular thrombosis is emerging as a significant public health concern in India, driven by a complex interplay of lifestyle, genetic predisposition, and socio-economic factors. This review examines recent trends in the prevalence, risk factors, and epidemiology of these conditions within the Indian population. A systematic search and selection process was employed to identify relevant studies, focusing on articles published between 2007 and 2024. Key findings indicate that rapid urbanization, dietary shifts, and increased stress levels contribute significantly to the rising rates of CVD and thrombosis. This review also highlights the critical comorbidity of mental health and CVD where mental health disorders exacerbate cardiovascular risks and complicate treatment outcomes.

Adopting healthy lifestyles, the early detection and diagnosis of intravascular thrombosis and its predisposing factors, timely prevention, increased public awareness about available treatments, accessibility to healthcare resources, and mental health integration are the keys to preventing mortality associated with cardiovascular diseases.

## Introduction and background

Cardiovascular diseases (CVDs) encompass a range of disorders affecting the heart and blood vessels, such as coronary artery disease (CAD), stroke (cerebrovascular disease), peripheral artery disease, rheumatic heart disease, congenital heart conditions, deep vein thrombosis (DVT), and pulmonary embolism (PE) [1]. Among these, intravascular thrombosis, characterized by the formation of blood clots within blood vessels, plays a critical role in the pathogenesis of cardiovascular events, such as myocardial infarction and stroke.

The population attributable risk (PAR) reflects the combined effect of the prevalence of risk factors and its associated risk in the population globally in percent for risk factors such as hypertension (24%), diabetes (10%), and dyslipidemia (39%) combined with lifestyle behaviors such as smoking (36%), excessive alcohol consumption (16%), tobacco use (1.9 million deaths from tobacco-related cardiovascular disease globally), inadequate physical activity (11%), and poor diet quality (12%); psychosocial factors such as personal and work-related stress and depression; and life events (32%) that enhance the risk of intravascular thrombosis and, consequently, cardiovascular events [2]. Additionally, genetic factors and socio-economic disparities further complicate the landscape leading to varied outcomes across different populations [3].

## Review

Methodology

A descriptive review was conducted to assess recent trends in cardiovascular disease (CVD), intravascular thrombosis, and their correlation with mental health within the Indian population. The search for relevant literature was carried out between 05/08/2024 and 30/08/2024 across multiple databases and sources, including PubMed, Springer, Scopus, Annals of Indian Academy of Neurology, Turkish Society of Cardiology, Indian Heart Journal, reputable websites such as the World Health Organization (WHO) website, National Family Health Survey-5 (NFHS-5) fact sheet, and articles published by the National Institute of Mental Health and Neurosciences (NIMHANS) and Cleveland Clinic. The search terms used included "cardiovascular disease", "intravascular thrombosis prevalence", "mental health and CVD", and "CVD trends in India". The inclusion criteria focused on articles published in English between July 2007 and April 2024 that examined studies conducted within the Indian population. Articles that were published before 2007, did not have free full text, contained irrelevant data, and were not published in English were excluded. After a thorough selection process, 16 articles were finalized for the review, excluding duplicates and articles that did not meet the criteria. The selection was based on the topic's relevance and the quality of the sources, such as those published in high-impact journals, ensuring that only the most pertinent research was included to comprehensively address the objectives of this review (Table 1).

**Table 1:** Criteria for literature search Source: [4] WHO, World Health Organization; NFHS-5, National Family Health Survey-5; NCDs, noncommunicable diseases; NIMHANS, National Institute of Mental Health and Neurosciences; CVD, cardiovascular disease

Items	Specification
Date of search	05/08/2024 to 30/08/2024
Databases and other sources	PubMed, Springer, Scopus, Annals of Indian Academy of Neurology, Turkish Society of Cardiology, Indian Heart Journal, WHO website, NFHS-5 fact sheet, presentation on "Mental health & NCDs- An Introduction" by NIMHANS, and Cleveland Clinic published article
Search terms	Cardiovascular disease, intravascular thrombosis prevalence, mental health and CVD, and CVD trends in India
Inclusion criteria	Related articles from July 2007 to April 2024, study conducted on Indian population, and articles published in English
Exclusion criteria	Articles published before 2007 and articles with no free full text
Selection process	Articles were selected based on the relevancy and quality from the abovementioned databases and other sources. Sixteen articles were finalized and were included in the review after excluding duplicates, articles with no full text, and irrelevant data
Any additional considerations	Five websites were also included

Epidemiology

CVD is a leading cause of mortality and morbidity globally, and India is no exception [2]. According to the Global Burden of Disease Study's (2010) age-standardized estimates, nearly a quarter (24.8%) of all deaths in India are attributable to CVD. The age-standardized CVD death rate of 272 per 100,000 populations in India is higher than the global average of 235 per 100,000 populations indicating that India has a higher burden of CVD [5].

There has been a significant rise in premature mortality due to CVD by 59% from 1990 (23.2 million) to 2010 (37 million). CVDs strike Indians a decade earlier than the Western population [5,6]. The most recent data published by the National Crime Records Bureau (NCRB) in its report "Accidental Deaths and Suicides in India" presents a concerning scenario, revealing that 32,457 people died from heart attacks in 2022, a notable increase from the 28,413 deaths reported in the previous year [7].

Studies also stated that by 2025, over five million premature CVD deaths in men and 2.8 million in women are estimated globally. The prevalence of coronary artery disease (CAD) has also shown a marked increase, rising from 1%-2% in urban populations in the 1960s to around 10%-12% in recent years. Similarly, rural areas have seen an increase in CAD prevalence from 2%-3% to 4%-6% during the same period. In 2016, India reported 63% of total deaths due to NCDs, of which 27% were attributed to CVDs. CVDs also account for 45% of deaths in the 40-69-year age group [1,8]. Age-specific years of life lost (YLL) per 100,000 populations according to the WHO report (2017-2018) starts high in infants under one year of age and decreases after attaining one year and increases again when one attains 40 years of age and much higher in 80 years and above [9]. Regional-wise, Tamil Nadu and Madhya Pradesh have the highest YLL, and it is lower in Jharkhand and Assam [1,10,11]. Interestingly, the report also showed that less-developed regions have lower CVD cases in comparison with developed regions, when measured through the Human Development Index (HDI) [1].

Disease burden

According to a WHO India Report, the age-adjusted CVD death rate is 349 per 100,000 in men and 265 per 100,000 in women [12-14]. According to the Global Burden of Disease Study, heart disease is the leading cause of death among women in India, accounting for almost 18% of all female deaths. Surprisingly, the death rate from heart disease among Indian women exceeds that of breast cancer and all other cancers combined [12,14].

Thrombosis, a critical component for many cardiovascular conditions, has also shown concerning trends. Venous thromboembolism (VTE), which encompasses deep vein thrombosis (DVT) and pulmonary embolism (PE), impacts an estimated 1-2 individuals per 1,000 each year in low- and middle-income countries (LMICs) [6]. The incidence of these conditions is rising due to the present medical conditions such as diabetes (10%-12%), hypertension (30%), dyslipidemia (25%-30%), sedentary lifestyles (41%), obesity (20%-25%), unhealthy diet (75%), and smoking and tobacco use (29%). Moreover, during pregnancy, women have high chances of developing thrombosis, which increases in the initial three months after delivery, the reason being the lack of physical activity, hormonal imbalance, and inflammation. Roughly one out of every 1,000 pregnancies in India is linked to VTE due to pregnancy [15].

Demographic variations also play a significant role in the epidemiology of CVD and thrombosis in India. Generally, men have a 2-3 times higher risk of developing CVD in comparison with women; the chances of getting venous thromboembolism are estimated to be 1.5-2 times greater in pre-menopausal women than in men and vice versa after women reach their menopausal stage [9,12]. Factors such as pregnancy, the use of oral contraceptives, and hormone replacement therapies significantly elevate this risk. Age is another critical factor, with a significant rise in CVD over the age of 40 [6].

"If there is a family history of CVD, then it is imperative to start regular check-ups and understand your risk factors by the age of 20. It's important to be practical; individuals with one or two known risk factors should consider having a comprehensive check-up between the ages of 40 and 55," advised Dr. Ashwin B. Mehta, director of cardiology at Jaslok Hospital & Research Centre and consultant cardiologist at Sir H. N. Reliance Foundation Hospital and Breach Candy Hospital in Mumbai [7]. The prevalence of hypertension as per the National Family Health Survey-4 (NFHS-4) was found to be 18.1% in 2015-2016, while 21% of females aged over 15 years had hypertension compared to 24% of males of the same age range, as estimated in NFHS-5 (2019-2021) [16]. NFHS-5 also found a significant increase in the incidence of newly diagnosed cases of hypertension among individuals aged 15-49 years at 52.06%, while in NFHS-4, the newly diagnosed cases were 41.65% of the same age cohort [17,18]. Although rates of hypertension are lower among adolescent women compared to adolescent men, rates are advanced in women and seniors [9,19].

Mental illness significantly adds to the CVD burden. Individuals with severe mental illnesses (SMI) such as schizophrenia and bipolar disorder have a 2-3 times higher risk of developing CVD, largely due to factors such as increased smoking, poor diet, inactivity, and side effects of medications. Common mental disorders (CMD) such as depression and anxiety, affecting 10%-15% of Indian adults, also raise CVD risk by 1.5-2 times. Depression, for example, is linked to inflammation and higher blood pressure, both risk factors for heart disease. These combined risk factors contribute to the complex relationship between mental health and CVD. The Global Burden of Disease Study estimates that mental health and stress-related illnesses, including stress-induced cardiovascular conditions, contribute to billions in lost productivity annually in India. The study also highlighted the growing concern of the comorbidity of CVD with mental health issues such as depression and anxiety (Table 2) [20].

**Table 2:** Interaction of cardiovascular disease (CVD), thrombosis, risk factors, and mental health across various settings in India Source: [20] SMI: serious mental illness

Risk factor/setting	CVD and thrombosis	Mental health condition	Causes of mortality
Person who smokes and is obese	Higher risk of CVD and thrombosis	Smoking and alcohol use in SMI	-
CVD and thrombosis (diabetes and hypertension)	Diabetes and hypertension linked to CVD and thrombosis	Diabetes and depression or SMI	-
Mental health condition	Mental health disorders exacerbating CVD risks	SMI with coexisting CVD and behavioral risks	-
Rural population, South India (schizophrenia)	Cardiovascular diseases (50%)	Schizophrenia with CVD	Cardiovascular (50%)
Hospital setting (45% with SMI)	Cardiovascular (43.6%) and thrombosis-related mortality	SMI exacerbating cardiovascular complications	Cardiovascular (43.6%)

Moreover, it was also found that the cause of mortality in patients with serious mental illness (SMI) is largely contributed by CVD [20].

Risk factors

Cardiovascular disease (CVD) and thrombosis are influenced by both lifestyle choices and genetic predispositions [21]. Dietary habits, such as a high intake of saturated fats, trans fats, and sodium, are leading contributors to dyslipidemia, hypertension, and obesity, all of which elevate more than 50% of CVD deaths [6,21]. Physical inactivity, especially in urban populations, further increases susceptibility to conditions such as insulin resistance [11,21,22]. Lifestyle factors such as smoking and tobacco use (29%) and excessive alcohol consumption (14%; men, 27%; women, 1.6%) significantly exacerbate the risk of CVD and thrombosis in India [2,5,6,17,21,22]. High body mass index (BMI) (24%-26%), aging (approximately 10.1%), and increased stress levels (around 74%), particularly in younger individuals, are also important risk factors [6,11]. These factors collectively contribute to the growing burden of cardiovascular conditions in India.

In addition to lifestyle risks, several medical conditions also contribute to the increasing prevalence of CVD and thrombosis. Diabetes mellitus (10%-12%), hypertension (30%), obesity (20%-25%), cancer treatments (20%), blood-clotting disorders (1%-5%), inflammatory conditions (0.02%-1%), and infections such as COVID-19 (30%-40%) also elevate the risk [3,5,6,8,13,17,19,21,22]. A family history of CVD or thrombosis also plays a significant role due to shared genetic and environmental influences. Additionally, hormonal factors such as birth control, hormonal therapy, and pregnancy can raise the risk of thromboembolism, making it crucial to consider non-estrogen contraceptive devices for those at high risk of blood clots [14,15].

Treatment gaps

Data from the Prospective Urban Rural Epidemiology (PURE) study indicates that up to three-fourths of patients with coronary artery disease (CAD) are not receiving the basic therapy drugs recommended by guidelines. This lack of adherence to treatment protocols is likely a significant contributor to the higher rates of morbidity and mortality observed in these patients [17]. Also, despite the global disease burden associated with the co-occurrence of cardiovascular diseases (CVDs) and depression, depression remains underdiagnosed and undertreated in the CVD population, especially among older adults in India, thereby increasing mortality in CVD patients [12,18].

Diagnosis

In the context of rising cardiovascular disease (CVD) and thrombosis in India, clinical diagnostics plays a critical role in identifying and managing conditions such as deep vein thrombosis (DVT) and pulmonary embolism (PE). Physical examination and patient history, such as recent surgeries, infections, or prolonged immobility, are essential in assessing thrombosis risk factors. Computed tomography pulmonary angiography (CTPA) is a vital diagnostic tool, offering high-resolution images of blood vessels to detect clots in the lungs. Blood tests such as the D-dimer test help evaluate clotting activity, while oxygen levels and cardiac and pulmonary functions provide insight into overall patient health. These diagnostic approaches are essential to early detection and treatment, crucial in curbing the growing burden of thrombotic diseases in India's evolving epidemiological landscape [15].

Treatments

The treatment of cardiovascular disease and thrombosis primarily involves medications such as blood thinners or anticoagulation therapy, which help prevent further clot formation. In critical cases, clot-dissolving medications may be administered intravenously to rapidly break down dangerous clots. For some patients, a catheter-assisted procedure may be required to physically remove or break up large clots. Additionally, in certain high-risk cases, a vena cava filter can be placed in the large vein to catch clots before they travel to the lungs, reducing the risk of severe complications such as pulmonary embolism [15].

Recovery

Recovery from cardiovascular disease and thrombosis requires routine follow-ups and strict adherence to prescribed medications to ensure effective management and prevent a recurrence. Addressing mental health and managing stress are crucial, as emotional well-being directly impacts physical recovery. Patients are encouraged to adopt healthy lifestyle changes, including consuming heart-healthy foods rich in vitamin K, such as leafy greens, soybean oil, and fruits, to support blood clotting and overall vascular health. Maintaining a healthy weight and abstaining from smoking or tobacco use are also critical for long-term recovery and reducing the risk of future cardiovascular events [15].

## Conclusions

Cardiovascular diseases and intravascular thrombosis are pressing health issues in India, driven by lifestyle factors, genetic predisposition, and socio-economic disparities. In India, CVD tends to affect individuals at younger ages compared to Western populations, leading to higher rates of premature mortality. Lifestyle changes, such as unhealthy diets, physical inactivity, and stress, contribute to the rising CVD prevalence, while mental health disorders such as depression and anxiety further complicate treatment, resulting in poor health outcomes. Gender also plays a significant role, with men generally at higher risk and women facing unique challenges linked to hormonal changes. Socio-economic disparities affect education and access to healthcare, leading to gaps in diagnosis and treatment. Addressing these issues requires a multifaceted approach involving early diagnosis, preventive measures, mental health integration, and lifestyle interventions, which are crucial for reducing the CVD burden in India. Enhancing public health awareness, promoting adherence to treatment protocols, and improving access to healthcare resources will be essential in curbing the growing epidemic of CVD and intravascular thrombosis in the coming years.
